# Treatment of aphasia in linguistically diverse populations: current and future directions

**DOI:** 10.3389/fpsyg.2025.1612413

**Published:** 2025-08-14

**Authors:** Gerald C. Imaezue, David Ajayi, Celine Davis

**Affiliations:** Department of Communication Sciences and Disorders, University of South Florida, Tampa, FL, United States

**Keywords:** aphasia treatment, multilingual aphasia therapy, restorative aphasia therapy, nonlinguistic aphasia therapy, AI-driven aphasia therapy

## Abstract

Aphasia is a multimodal language disorder that affects individuals across all language cultures, disrupting speaking, listening, reading, writing, and gestural communication. Although aphasia is challenging to manage in monolingual individuals, it becomes even more complex in linguistically diverse populations due to factors such as differences in language-specific features, limited linguistically customizable behavioral therapies and clinicians’ cross-linguistic competence. This critical review examines current and emerging treatment approaches for linguistically diverse populations, highlighting the progression from traditional behavioral interventions to innovative modalities, including state-of-the-art AI-driven and culturally sensitive interventions designed to overcome cultural and linguistic barriers and enhance therapy outcomes. The review emphasizes the growing need for aphasia care specific to linguistically diverse populations, with a focus on personalized treatment strategies and innovations in digital therapeutics that account for cultural and linguistic nuances. Specifically, we advocate for: (1) personalizing restorative aphasia therapies to users’ preferred languages; (2) restorative therapies that leverage universal nonverbal systems and neurobiological modulations as primary treatment modalities; and (3) digital innovations such as multilingual artificial intelligence systems for restorative aphasia therapy, particularly delivered through smartphones. Additionally, ethical considerations—including cultural responsiveness, clinician preparedness, and patient data protection-are discussed to inform future directions in equitable and effective aphasia care. Overall, this study provides insights to guide the development of inclusive and innovative aphasia interventions for linguistically diverse populations.

## 1 Introduction

### 1.1 Aphasia, classification and prevalence

Aphasia is a multimodal language disorder that impairs expressive and receptive language skills and other communication modalities, such as speaking, auditory comprehension, writing, reading and gestural communication (Fridriksson and Hillis, 2021). Its severity, presentation and prognosis for recovery vary and are influenced by the interaction of complex intrinsic factors such as the site of lesion, extent of brain damage, time post onset, and extrinsic factors like treatment use, relevance and accessibility (Fridriksson and Hillis, 2021; Plowman et al., 2012). Stroke remains the leading cause of aphasia, as it accounts for at least a quarter of aphasia cases (Grönberg et al., 2022). However, other conditions such as traumatic brain injury, brain tumor and neurodegenerative diseases could lead to aphasia. The National Aphasia Association (NAA) estimates that aphasia affects over two million individuals in the United States (Breining and Sebastian, 2020). Aphasia is a significant public health concern due to its adverse impact on functional communication, mobility, and overall quality of life (Bueno-Guerra et al., 2024).

Our study focuses on stroke-induced aphasia. Aphasia is broadly categorized into fluent and nonfluent subtypes. Fluent aphasia, like Wernicke’s aphasia or conduction aphasia, is characterized by relatively intact speech production with impaired language comprehension and paragrammatic speech: frequent word substitution errors or word misuse during spontaneous speech (Cordella et al., 2024). Nonfluent aphasia, such as Broca’s aphasia or transcortical motor aphasia, involves effortful, halting, fragmented, and agrammatic speech–omission of grammatical elements, with relatively preserved comprehension. Some persons with aphasia (PWA) exhibit severe production and comprehension deficits concurrently, a condition clinically defined as global aphasia (Sheppard and Sebastian, 2021).

### 1.2 Linguistic diversity in aphasia

Aphasia is a heterogeneous disorder, presenting diverse patholinguistic profiles even among monolingual speakers. These complexities are further amplified in multilingual individuals with aphasia and linguistically diverse populations. Multilingual aphasia specifically refers to cases where individuals acquired and use two or more languages before the onset of aphasia. The assessment and treatment of this condition requires careful consideration of factors such as age of language acquisition, pre-and post-stroke as well as patterns of language use (Ansaldo et al., 2014; Goral and Lerman, 2020). In contrast, aphasia in linguistically diverse populations refers more broadly to individuals who may or may not be multilingual but who reside in multilingual societies or belong to linguistic minority groups (Arslan and Penaloza, 2025). For these individuals, extrinsic factors such as lack of linguistically appropriate treatments and access to multilingual clinicians can pose significant barriers to aphasia care (Goral and Lerman, 2020).

Overall, treatment of aphasia in linguistically diverse populations is limited by differences in language-specific features across cultures which then lead to lack of culturally and linguistically appropriate assessment tools, limited linguistically customizable behavioral therapies and shortage of clinicians with cross-linguistic competence. For instance, most standardized tests are designed for monolingual speakers, making them less accurate for individuals from minority language backgrounds (Noroozi et al., 2021). Access to clinicians who speak the patient’s language is also limited, which can affect communication, engagement, and treatment outcomes. In multilingual aphasia, factors like language dominance, proficiency differences, and cross-linguistic interference further complicate diagnosis and therapy planning, which must account for syntactic, phonological, and semantic variation across languages. As global migration increases, there is a growing need for aphasia treatments that are linguistically and culturally responsive (Ansaldo et al., 2014; Centeno and Harris, 2021; Centeno and Laures-Gore, 2024). Meeting this challenge will require the development of language-agnostic and culturally tailored interventions delivered using ubiquitous technologies such as smartphones, and AI-driven multilingual technologies designed to support equitable and effective aphasia rehabilitation worldwide.

### 1.3 Aims of the study

This critical narrative review aims to evaluate the current landscape of aphasia treatments in linguistically diverse populations, explore innovative approaches such as personalized and technology-assisted therapies and their applications in multilingual contexts, and discuss strategies for developing linguistically inclusive and accessible treatments for multilingual patients with aphasia. To achieve these objectives, we first examine contemporary treatment approaches for aphasia in monolingual populations and consider their implications for diverse linguistic groups. We then review non-linguistic cognitive strategies that may help overcome language barriers in cross-linguistic settings, and finally, we highlight recent advances in artificial intelligence (AI) and multilingual speech and language technologies with the potential of driving personalized therapies for linguistically diverse populations.

## 2 Current restorative treatments for aphasia

### 2.1 Restorative behavioral aphasia therapies

Contemporary approaches to aphasia therapy primarily involve behavioral interventions aimed at restoration of impaired language functions or enabling compensatory strategies for functional communication, with both approaches having implications for improving quality of life (Sheppard and Sebastian, 2021). Restorative aphasia therapy is a key behavioral intervention that plays a crucial role in language recovery by using linguistic tasks to actively target and engage specific impaired linguistic systems, leveraging principles of use-dependent learning and neuroplasticity as well as psycholinguistic and neurolinguistic theories of language processing. Restorative aphasia therapies are recognized as the gold standard for aphasia treatment, employing evidence-based techniques to minimize aphasic speech and language errors and ultimately facilitating PWA’s functional communication abilities. These interventions can be broadly categorized based on linguistic domain (e.g., production or comprehension), linguistic level (e.g., word, sentence or discourse), and modality (e.g., spoken, written, auditory or reading).

To address difficulty with spoken language production at the word level, therapies such as Semantic Feature Analysis (SFA) and Phonological Component Analysis (PCA) have been widely used. SFA enhances lexical retrieval by training persons with aphasia to generate and reflect on semantic attributes related to target words, thereby strengthening the semantic network and aiding access to word meaning (Bihovsky et al., 2024; Efstratiadou et al., 2018; Boyle and Coelho, 1995). In contrast, PCA emphasizes phonological characteristics such as syllable structure and initial sounds, enhancing phonological encoding during word retrieval (Leonard et al., 2008; Meteyard and Bose, 2018; Python et al., 2025). These therapies are particularly beneficial for individuals with anomic aphasia or other naming deficits, helping to reduce word-finding difficulties. At the sentence level of spoken language production, approaches like Melodic Intonation Therapy (MIT), Constraint-Induced Language Therapy (CILT) and script training have been used to improve language production in PWA. MIT uses two distinct pitches (high and low) to create melodic speech engaging the relatively preserved right hemisphere to help compensate for impaired left-hemisphere networks (Albert et al., 1973; Conklyn et al., 2012; Norton et al., 2009; Haro-Martínez et al., 2019). CILT, on the other hand, promotes use-dependent learning by restricting the use of compensatory non-verbal modalities (e.g., gestures or writing), thereby forcing reliance on spoken language and encouraging functional sentence production through intensive practice (Meinzer et al., 2005, 2007; Pulvermuller et al., 2001; Raymer and Roitschb, 2023). Script training emphasizes the automatic production of script-based sentences to facilitate functional communication (Cherney et al., 2008; Cherney and Van Vuuren, 2022; Quique et al., 2022; Youmans et al., 2005).

Although language production-focused therapies are well represented in the literature, comprehension-based restorative therapies are equally essential. These interventions often aim to improve auditory or written language comprehension through activities such as sentence-picture matching, syntactic mapping, or treatment of underlying forms, depending on the individual’s comprehension profile (Fleming et al., 2021; Thompson, 1996; Thompson and Shapiro, 2005; Swiderski et al., 2021; Wallace et al., 2022; Webster et al., 2013). These techniques help improve communication by engaging language-dominant and compensatory neural pathways to induce language recovery in PWA.

### 2.2 Restorative neuromodulation and pharmacological therapies

Neuromodulation in aphasia treatment consistently involves the use of noninvasive methods aimed at exciting or inhibiting neural activity in target regions of interest to enhance aphasia recovery. Among noninvasive brain stimulation (NIBS) techniques, repetitive transcranial magnetic stimulation (rTMS) and transcranial direct current stimulation (tDCS) are the most widely studied (Breining and Sebastian, 2020; Gao et al., 2019; Williams et al., 2024). NIBS techniques are primarily used as adjuvants to behavioral aphasia therapy, targeting ipsilesional, perilesional or contralesional language brain regions as well as nonconventional regions like the cerebellum or primary motor cortex to induce language recovery in aphasia (Baker et al., 2010; Chrysikou and Hamilton, 2011; Crosson et al., 2019; Darkow et al., 2017). Studies have shown consistently that pairing restorative language therapy with rTMS or tDCS leads to better language outcomes in patients with nonfluent aphasia than using NIBS alone (Allendorfer et al., 2021a; Breining and Sebastian, 2020; Fridriksson et al., 2018; Raymer and Johnson, 2024). Although the optimal parameters and mechanism of action underpinning the effect of NIBS-induced aphasia recovery remains unclear, studies have consistently shown that inhibition of the contralesional right hemisphere and excitation of ipsilesional or perilesional language regions are optimal for driving language recovery (Allendorfer et al., 2021b; Marangolo et al., 2016; Williams et al., 2024) but see Turkeltaub (2015) for discussions on contradictory evidence regarding the role of contralesional regions in aphasia recovery.

Pharmacological interventions are used to modulate impaired neurochemical pathways to drive language recovery in aphasia. Like studies on neuromodulation in aphasia care, no studies have conclusively shown that pharmacological interventions alone led to better language recovery in PWA than when combined with restorative behavioral aphasia therapy (Saxena and Hillis, 2017). For instance, evidence from randomized controlled trials suggests that pharmacological agents such as donepezil and memantine may enhance naming, oral expression, auditory comprehension, and repetition in post-stroke aphasia, particularly when combined with restorative behavioral treatments (Cichon et al., 2021; Fridriksson and Hillis, 2021).

While these findings demonstrate the effects of neuromodulation and pharmacological interventions as adjuvants to restorative behavioral aphasia therapy, further studies are needed to establish their efficacy and clinical applicability, especially in diverse cross-linguistic populations.

### 2.3 Linguistic barriers to restorative aphasia therapies

Restorative therapies for aphasia including behavioral, neuromodulation, and pharmacological interventions have been predominantly studied in speakers of Indo-European languages, particularly English. This linguistic narrowness presents a critical limitation in global aphasia care, as aphasic symptoms do not manifest uniformly across languages. Structural differences among languages can shape both the nature of aphasia and its response to treatment. For instance, languages with rich inflectional morphology, such as Italian or German, often elicit different error patterns (e.g., more substitutions) than less morphologically complex languages like English, which tend to show more omissions (Kiran and Roberts, 2010). These differences necessitate language-specific therapeutic approaches. Clinicians must therefore tailor assessments and interventions to the grammatical and lexical features of each language, accounting for factors such as word order, subject pronoun usage, and verb inflection, rather than applying English-based protocols uncritically.

Restorative aphasia therapies rely heavily on dyadic verbal interactions, which can be compromised when speech-language pathologists (SLPs) do not speak the patient’s primary language (Larkman et al., 2024). This linguistic mismatch risks compromising the validity of assessments, reducing patient engagement, and limiting therapeutic outcomes. Moreover, most evidence-based treatment protocols have been designed and validated in English, offering limited guidance for their adaptation to non-English-speaking or culturally diverse populations. Given that over 7,000 languages are spoken globally (Karim and Sima, 2015), and that stroke-induced aphasia occurs across all linguistic and cultural groups, the lack of cross-linguistic generalizability poses a significant barrier to equitable and effective aphasia care.

Restorative therapy accessibility is particularly uneven across language groups, presenting a profound obstacle to universal aphasia care. For instance, English-speaking individuals benefit from a broad array of standardized aphasia tests, treatment resources, and trained clinicians. In contrast, speakers of minority, indigenous and other languages often lack equivalent linguistically-sensitive therapies, normative data-driven assessments, access to cross-linguistically competent clinicians and professional support (Ansaldo et al., 2008; Ansaldo et al., 2014; Goral and Lerman, 2020; Ivanova and Hallowell, 2013). Multilingual patients with aphasia face unique challenges, including variable proficiency across their spoken languages—a factor often overlooked in conventional therapy approaches (Goral et al., 2023). This disparity means that many individuals must undergo assessment and treatment in a second or majority language, increasing the risk of misdiagnosis and culturally incongruent aphasia care (Centeno and Ansaldo, 2016). Speakers of under-resourced languages are especially vulnerable, as linguistic and cultural barriers have been shown to impede access to comprehensive aphasia services (Centeno, 2009). Although the mechanism of action of some behavioral therapies may have cross-linguistic applicability, e.g., semantic feature analysis (Bihovsky et al., 2024; Kiran and Roberts, 2010), there is no study that have established universal applicability of restorative aphasia treatment. Hence, the global shortage of linguistically appropriate assessments and culturally trained professionals thus limits the scalability and inclusivity of restorative therapies (Arslan and Penaloza, 2025; Centeno and Harris, 2021; Gobbo and Marini, 2024).

Addressing these linguistic and cultural gaps is essential for achieving equitable outcomes in aphasia rehabilitation. Recent scholarship has emphasized the urgent need to develop aphasia assessments in a wider range of languages and to validate existing protocols cross-linguistically (Ivanova and Hallowell, 2013). In parallel, training programs must prepare SLPs to deliver therapy that is not only linguistically but also culturally responsive. Without these efforts, the reach and effectiveness of aphasia therapies will remain restricted to a subset of the global population. Expanding resources beyond hegemonic languages and using language-agnostic tools are therefore prerequisites for ensuring that restorative aphasia therapies are accessible, relevant, and effective for all individuals affected by this disabling condition.

To address these critical needs, we further advocate for: (1) customizing restorative behavioral aphasia therapies to users’ preferred languages; (2) restorative therapies that leverage universal nonverbal systems and neurobiological modulations as primary treatment modalities; and (3) digital innovations such as multilingual artificial intelligence systems for restorative aphasia therapy delivered through smartphones.

## 3 Customizing aphasia therapy to users’ preferred languages

### 3.1 Importance of language preference

Language preference is crucial in aphasia therapy for multilingual patients. Research shows that strategies like targeting dominant languages, alternating treatment languages, focusing on shared linguistic structures, using patients’ preferred languages, and incorporating culturally relevant materials improve engagement, neuroplasticity, and recovery in both trained and untrained languages (Ansaldo et al., 2008; Edmonds and Kiran, 2006; Goral et al., 2023; Kiran and Iakupova, 2011; Kiran and Thompson, 2019; Kuzmina et al., 2019; Mooijman et al., 2024). Multilingual patients with aphasia often experience greater preservation and better performance in their first language (L1) compared to later learned languages. Correspondingly, delivering therapy in the preferred language tends to yield greater recovery gains. For example, Scimeca et al. (2024) found that multilingual patients who received naming therapy in their L1 showed significantly larger improvements in their first language compared to those treated in their second language. Allowing patients to use the language they are most comfortable with also enhances engagement in therapy. Studies have demonstrated that when multilingual patients with aphasia were free to switch between languages at will (reflecting their natural communication preference), they achieved faster and more accurate naming performance in single language conditions (Mooijman et al., 2024). This suggests that respecting a multilingual person’s language choice and permitting code-switching can reduce frustration, enhance participation, and leverage all available communicative resources for rehabilitation. Furthermore, tailoring aphasia therapy to a patient’s linguistic background and preference has been shown to improve rehabilitation outcomes, particularly, for instance, by addressing syntactic and phonological differences between languages thereby making treatment more linguistically relevant for patients (Edmonds and Kiran, 2006).

Rehabilitation in a patient’s preferred or most dominant language pre-stroke may optimally engage intact neural networks and facilitate reorganization. In multilinguals, the language that recovers better is often the most used and the one with stronger pre-stroke connections to cognitive control networks, underscoring the benefit of focusing therapy on the more proficient language (Kuzmina et al., 2019). Additionally, engaging PWA in their preferred language may enhance patient engagement, support recovery, and potentially improve generalization of therapeutic gains across different languages, particularly in cases where the later-learned language (L2) has become the primary language used for everyday conversation (Goral et al., 2023).

In summary, recent evidence from the past decade strongly supports tailoring aphasia therapy to the multilingual person’s language preference, as this approach maximizes therapy engagement, encourages adaptive neuroplasticity, and ultimately improves communication outcomes.

### 3.2 Use of interpreters and cultural mediators

In clinical practice and community-based settings, language interpreters and cultural mediators are vital for delivering effective cross-linguistic aphasia therapy to multilingual patients with aphasia especially when their languages differ from those spoken by clinicians. Professional interpreters facilitate accurate two-way communication, ensuring that clinicians can understand patients’ responses and patients can fully comprehend therapy instructions, preventing misunderstandings that could hinder therapy (Larkman et al., 2024). This accuracy is crucial in aphasia, where nuances of language ability need to be precisely gauged; studies have shown that miscommunications (e.g., omissions or alterations of patient responses) can impede diagnosis, goal-setting and cause emotional detachment from the therapeutic process underscoring the need for interpreter training in aphasia care (Babbitt et al., 2022; Larkman et al., 2023).

Equally important, cultural mediators (sometimes called cultural or language brokers) help tailor interventions to the patient’s cultural context, bridging gaps in understanding and building trust between patients and providers. Clinicians are advised to collaborate with both interpreters and cultural mediators to maximize therapy engagement and relevance for multilingual patients. Cultural mediators often take on multiple functions in these settings, acting as patient advocates and navigators who connect individuals and families with resources in a culturally sensitive manner (Sharma et al., 2023). For instance, mediating cultural differences by explaining rehabilitation practices in the light of patient’s cultural differences and adjusting activities to be culturally meaningful could make interventions culturally relevant, ultimately enhancing patient’s motivation and adherence to therapy activities. Such culturally responsive approaches have been shown to improve therapy engagement and satisfaction, as patients feel understood and respected in their values and communication styles (Sharma et al., 2023). Conversely, the absence of such support is associated with disparities; for example, multilingual patients with aphasia who required interpreters have been found less likely to receive certain evidence-based language interventions and had longer rehabilitation stays, indicating the risk of poorer outcomes without language assistance (Mellahn et al., 2025). This evidence highlights that interpreters and cultural mediators are not just add-ons but essential contributors to equitable and effective clinical aphasia care.

Overall, the combined efforts of interpreters and cultural mediators help ensure that patients with aphasia in cross-linguistic settings receive support that is linguistically accurate and culturally appropriate, leading to significantly long-term recovery and improved quality of life.

### 3.3 Aphasia assessment in multicultural settings

Although this paper focuses on restorative aphasia treatments, accurate assessment of language proficiency and dominance pre-and poststroke is critical for developing customized treatment plans for patients with aphasia in linguistically diverse settings. Tools such as the Bilingual Aphasia Test (BAT) (Paradis and Libben, 2014), the Language Experience and Proficiency Questionnaire (LEAP-Q) (Marian et al., 2007) and the Bilingual Switching Questionnaire (BSWQ) (Rodriguez-Fornells et al., 2012) provide structured ways to evaluate a patient’s language history, dominance, and proficiency across languages. Dynamic formal and informal assessment methods, which assess language skills especially in real-time contexts, provide clinicians with a comprehensive understanding of a patient’s communicative abilities (Doedens and Meteyard, 2020; Salter et al., 2006).

Most reported standardized aphasia assessments are designed for monolingual English-speaking populations, limiting their utility in multilingual or culturally diverse contexts. Adapting these tools involves translating and culturally modifying assessment items to ensure relevance and fairness. For instance, the Boston Diagnostic Aphasia Examination (Goodglass et al., 2001); the Western Aphasia Battery-Revised (Kertesz, 2007) and the Bilingual Aphasia Test (Paradis and Libben, 2014) have been adapted for multiple languages, but these versions must consider cultural factors, such as the cultural validity and familiarity of test stimuli (Centeno and Laures-Gore, 2024). Clinicians are encouraged to employ dynamic assessments using culturally appropriate stimuli alongside standardized tools, if available, to capture nuanced linguistic abilities in underrepresented languages.

## 4 Toward universal restorative aphasia therapy

### 4.1 Recruiting nonlinguistic cognitive processes for language recovery

The rationale behind recruiting universal cognitive systems in aphasia rehabilitation is particularly significant when considering patients from diverse linguistic backgrounds. The fundamental cognitive processes that underpin language processing, such as attention, executive function and working memory, are believed to be universal across cultures. A growing body of research consistently supports the complex relationship between language, language recovery and nonlinguistic cognitive processes in adults with and without aphasia (Albert et al., 1973; Diedrichs et al., 2022; Dignam et al., 2017; Gilmore et al., 2019; Lambon Ralph et al., 2010; Seniów et al., 2009). Meta-analyses of neural activity in patients with aphasia performing language tasks reveal that their brains recruit regions beyond the traditional language networks (LaCroix et al., 2021). These include areas involved in domain-general cognitive processes across both cerebral hemispheres, with activation patterns similar to those of healthy controls (LaCroix et al., 2021). These insights suggest that therapies targeting these relatively preserved cognitive systems could enhance language recovery by leveraging relatively intact cognitive processes in aphasia patients, thus effectively addressing linguistic barriers in aphasia care.

A growing body of empirical evidence has explored the effectiveness of nonlinguistic cognitive training, often administered in conjunction with traditional aphasia therapy, in facilitating aphasia recovery particularly in stroke survivors. Studies, including a network meta-analysis evaluating various cognitive training interventions, found that combining computer-assisted cognitive training, conventional cognitive training, virtual reality-based cognitive training, telerehabilitation computer-assisted cognitive training, cognitive stimulation training, working memory training, or attention training with traditional aphasia therapy improved language outcomes in patients with aphasia than traditional aphasia therapy alone (Kong et al., 2024). Studies have further demonstrated that the incorporation of gradual attention training and programs targeting executive functioning alongside conventional aphasia therapy led to improvements in a range of language abilities, including reading comprehension, auditory comprehension, verbal sentence production and comprehension, naming, speech repetition, functional communication, and verbal fluency (Culicetto et al., 2025).

The studies discussed combined cognitive training with conventional aphasia therapy, which may be limited by linguistic diversity. Another research, while not focused on cognitive training, explores the use of intrinsic self-feedback mechanisms—specifically recursive self-feedback (RSF) for aphasia recovery. RSF is a self-directed automated procedure that enables individuals to iteratively monitor, evaluate, and adjust their own spoken output—typically by listening to recordings of their own speech and providing self-correction or elaboration at each cycle—without relying on external cues or clinician guidance (Imaezue et al., 2023). RSF has shown promise in improving language outcomes, including sentence production, spontaneous speech and target language production in both monolingual and multilingual PWA (Imaezue, 2024a; Imaezue et al., 2024b; Imaezue et al., 2025). Intrinsic self-feedback mechanisms, such as error monitoring and correction, may function independently of specific linguistic features, potentially making RSF a language-independent approach to aphasia care (Imaezue, 2024a, Imaezue et al., 2024b). RSF is a promising and useful procedure in the toolbox of clinicians to meet the language needs of PWA in diverse linguistic populations. However, the evidence-based for this technique is based on few PWA which suggests that more research is needed to test the hypothesis of universal applicability of this procedure for aphasia care. By targeting fundamental intrinsic-cognitive feedback mechanisms, RSF may offer a more universally applicable approach to rehabilitation than cognitive training combined with conventional therapy.

### 4.2 Multisensory stimulation for language recovery

Language barriers can significantly hinder the effectiveness of behavioral aphasia treatment, particularly when therapy relies heavily on verbal instructions. Multisensory stimulation offers a viable solution by engaging multiple nonverbal sensory modalities and neural pathways to support language recovery. Incorporating visual, auditory, tactile, and proprioceptive cues reduces reliance on verbal communication, enhancing accessibility and reinforcing learning (Diedrichs et al., 2022; Schuell et al., 1955).

Multisensory techniques transcend linguistic and cultural differences because they primarily rely on fundamental sensory and motor experiences rather than language-specific structures. Visual cues, gestures, facial expressions, and tactile feedback are universally understood and may not require proficiency in a particular language. For example, gesture-based cueing, such as pointing or hand movements that mimic actions or iconic gestural description of objects conveys meaning across cultures and can aid patients with fluent and nonfluent aphasia across cultures in understanding and expressing themselves (Attard et al., 2013; Rose et al., 2016; Wadams et al., 2022). Although multisensory techniques are often considered adaptable across linguistic and cultural contexts, particularly those involving gestures, gesture-based cues such as pointing and facial expressions may not always be interpreted uniformly across cultures and should therefore be applied with cultural sensitivity and individualized consideration (Efron, 1941; Noroozi et al., 2021).

A longstanding debate in aphasia rehabilitation concerns whether nonverbal and multisensory treatments can generalize to improvements in language such as spontaneous speech, which is a key marker of functional communication in real-world contexts. Some studies report little to no direct generalization to spontaneous speech following gesture-based interventions (Marshall et al., 2012; Marshall et al., 2013; Clough and Duff, 2020). In contrast, other studies suggest that nonverbal treatments can support language recovery indirectly, by improving naming or functioning as compensatory strategies, especially when used within a scaffolding framework, where gestures are gradually withdrawn as verbal abilities return (Rose et al., 2002; Rose et al., 2013; Clough and Duff, 2020). Additionally, multisensory approaches that pair speech with visual or motor cues, such as speech-associated gestures, have been shown through neuroimaging to reduce cognitive effort and increase neural activations during language production, likely by engaging shared motor-language networks (Skipper et al., 2007). Collectively, these findings suggest that while nonverbal treatments may not always lead to direct gains in language tasks like spontaneous speech, their integration into multisensory, individualized therapies could enhance functional communication and support language recovery post-stroke.

Similarly, rhythmic and melodic elements in therapy, such as those used in MIT, tap into music’s cross-cultural potential to bypass linguistic barriers while facilitating speech production (Albert et al., 1973; Sparks and Holland, 1976; van der Meulen et al., 2014; Van der Meulen et al., 2016; Haro-Martínez et al., 2019; Gu et al., 2024). The adaptability of MIT has been demonstrated in tonal languages such as Mandarin (Gu et al., 2024) and syllable-timed languages like Spanish (Haro-Martínez et al., 2017), highlighting its flexibility across diverse prosodic systems. However, while music engages broadly shared cognitive and emotional systems, it is important to acknowledge that musical structures, such as scales, rhythm, and harmonic conventions, vary significantly across cultures and may affect therapeutic resonance and familiarity (Shapiro, 2005).

Likewise, visual supports such as culturally appropriate visual scene cues and pictorial representations can offer nonverbal context without requiring verbal explanation, supporting communication across language boundaries (Cohn, 2020). Yet, pictorial systems are not inherently universal; visual conventions, symbolism, and narrative structure are culturally mediated and may influence how such stimuli are interpreted (Čeněk and Čeněk, 2015; Cho and Ishida, 2011). Taken together, these findings underscore the need to select and adapt musical and visual materials with cultural sensitivity. When tailored to the individual’s cultural and aesthetic background, these modalities can serve as effective tools for enhancing engagement, scaffolding verbal expression, and supporting therapy in cross-linguistic and-cultural settings.

Furthermore, combining auditory repetition with visual semantic cues for aphasia therapy has been shown to significantly improve naming abilities in aphasic patients (Rose et al., 2016). The use of visual motor imagery and action observation, based on mirror-neurons and frontoparietal mechanisms, have been shown to facilitate language outcomes in patients with aphasia by engaging multiple sensory modalities simultaneously, thereby promoting aphasia recovery (Lee et al., 2010; Rizzolatti and Craighero, 2004).

By leveraging these universally accessible sensory modalities, multisensory and nonverbal-based techniques ensure that treatment remains effective and adaptable for patients with aphasia from diverse cultural and linguistic backgrounds. Further research is suggested to explore this idea.

### 4.3 Neurobiological approaches as primary treatment modalities

Behavioral aphasia therapy has firmly established its role as an effective intervention for aphasia recovery (Breitenstein et al., 2017; Robey, 1998). However, the emerging evidence surrounding NIBS and pharmacological interventions presents a compelling case for their potential to evolve beyond their current status as adjunctive therapies in aphasia rehabilitation (Raymer and Johnson, 2024). The mechanisms of action of NIBS in promoting neuroplasticity through direct neural modulation and the capacity of pharmacological agents to modulate neurochemical pathways suggest a future where these neurobiological approaches could take on new roles as primary treatment modalities with greater potentials for worldwide applications than conventional behavioral therapies which are constrained by linguistic barriers. To attain this shift, however, several critical advancements are necessary. Continued rigorous research is essential to optimize NIBS protocols, including refining and personalizing stimulation parameters, developing more precise targeting strategies, and determining the optimal timing for intervention relative to the onset of aphasia. For instance, integrating neuroimaging and genomics into aphasia rehabilitation allows identifying neural and genetic markers associated with language recovery, which may help tailor pharmacological, NIBS and behavioral interventions to enhance individual treatment outcomes (Fridriksson et al., 2018; Kristinsson and Fridriksson, 2022; Shah-Basak et al., 2020; Wang et al., 2014).

Extensive and well-controlled clinical trials are needed to definitively establish the efficacy and safety of promising and novel pharmacological agents, as well as to determine the most effective ways to combine them with behavioral therapies for synergistic benefits. The development of personalized treatment strategies, guided by individual patient characteristics, detailed neuroimaging data, and the specific nature of their language impairments, will be crucial for maximizing the effectiveness of both NIBS and pharmacological interventions (Gkintoni and Michou, 2024; Saxena and Hillis, 2017). Technological advancements, particularly in the realm of advanced neuroimaging techniques and the development of artificial intelligence (AI)-driven personalized neurobiological treatment protocols, will play a significant role in facilitating this transition. Furthermore, advancements in stem cell therapy may have implications for neural repair poststroke and aphasia recovery in cross-linguistic populations (Baker et al., 2019). Addressing these current limitations and knowledge gaps through focused research and continued innovation is essential to fully unlock the potential of neurobiological interventions and transform the future of universal aphasia care.

In this evolving landscape, behavioral therapies could transition to a complementary role, focusing on functional communication and the generalization of gains achieved through neurobiological modalities. The realization of this future holds the promise of a paradigm shift in aphasia care, offering the potential for more effective and comprehensive rehabilitation strategies that address the underlying neurological basis of this debilitating condition while also leveraging the crucial contributions of behavioral therapies.

## 5 Digital innovations for accessible aphasia care

### 5.1 Smartphone-based aphasia therapy for accessible care

In response to global challenges like the COVID-19 pandemic, speech-language therapy shifted from in-person services to telehealth due to restrictions on in-person healthcare delivery. Telehealth emerged as an effective alternative, enabling patients with aphasia to continue receiving timely and consistent therapy from clinicians primarily through video conferencing platforms and other remote communication technologies (Dekhtyar et al., 2020; Hill et al., 2009; Taiebine and Keegan, 2024). This shift has also expanded access to care for multilingual and culturally diverse populations by facilitating connections with bilingual clinicians, integrating real-time translation and captioning tools, and allowing therapy to be tailored to individual linguistic needs, albeit across limited languages (Penaloza et al., 2021).

Telehealth-based aphasia therapy commonly relies on desktop or laptop computers, which may pose accessibility challenges (Brandenburg et al., 2013; Raymond et al., 2024). Many patients with aphasia, particularly those with co-occurring motor impairments, may find these devices difficult to use due to their size and the need for fine motor control. Additionally, desktops and laptops are less portable and may not support the flexible, frequent practice sessions that are essential for aphasia recovery. This highlights the need for mobile health (mHealth) solutions, particularly smartphone-based therapy, which offers a more accessible, portable, and user-friendly alternative with broader worldwide reach. Smartphones are widely available, affordable, and can be equipped with speech-language therapy apps, voice-activated controls, and adaptive accessibility features that can facilitate self-directed practice and clinician-guided interventions. The convenience afforded by the portability of smartphones allows patients with aphasia to engage in therapy from virtually any location at a time that suits their schedule, potentially leading to improved language outcomes, accessibility, adherence and a reduced burden on both patients and caregivers (Braley et al., 2021; Brandenburg et al., 2013; Imaezue and Goral, 2025; Jiang et al., 2024). By leveraging mHealth technologies, smartphone-based aphasia therapy presents a compelling practical avenue for addressing the limitations of traditional telehealth and enhancing inclusivity worldwide and responsiveness to the diverse needs of patients with aphasia (Jiang et al., 2024). However, smartphone-based interventions are not without limitations. The small screen size, variable hardware quality and device accessibility, poor user interface and experience design, word retrieval focused tasks, and the need for app-specific familiarity may pose accessibility, usability and engagement challenges for some users (Brandenburg et al., 2017; Greig et al., 2008; Nichol et al., 2021; Pontus et al., 2025; Vaezipour et al., 2020). Moreover, reliable internet access and device compatibility can vary across regions, potentially limiting scalability in low-resource contexts (Li and Wilson, 2013; McCool et al., 2022).

A growing number of specialized and evidence-based aphasia therapy mobile apps (e.g., Constant Therapy, Lingraphica and Tactus Therapy) are now available for smartphones, providing a rich array of tools for addressing various aphasia deficits. These apps often incorporate therapeutic techniques and exercises designed by SLPs, offering structured and engaging opportunities for patients with aphasia to practice their communication skills outside of formal therapy sessions (Nikolaev and Nikolaev, 2022; Vaismoradi et al., 2024). Notably, many of these platforms support multiple languages: Constant Therapy is available in English (US, India) and Spanish; Lingraphica’s Talk App supports dual language mode (English and Spanish); and Tactus Therapy offers full or partial translations in up to ten languages, including French, German, Dutch, and Zulu. This multilingual accessibility significantly enhances global reach and ensures that individuals with aphasia from linguistically diverse backgrounds can engage with therapy materials in their preferred or native language.

Studies have demonstrated the benefits of smartphone-delivered RSF for both scripted-sentence production and spontaneous speech in linguistically diverse individuals with aphasia (Imaezue, 2024a; Imaezue, 2024b; Imaezue et al., 2023; Imaezue et al., 2024b). RSF involves repeated playback and rehearsal of the patient’s own speech, enabling users to refine their output using intrinsic self-monitoring and correction mechanisms rather than external cueing. This approach is particularly advantageous for linguistically diverse populations because it uses the patient’s own speech as the therapeutic stimulus. As a result, RSF inherently preserves the speaker’s native accent, dialect, and phonological patterns. This is especially relevant given that accent, dialectal variation, and prosodic features can significantly influence speech intelligibility and therapeutic engagement—factors that are often underrecognized in conventional aphasia treatments (Goral and Hejazi, 2021; Mooijman et al., 2024). By bypassing the need for externally modeled stimuli, RSF ensures that feedback is linguistically and culturally congruent, which may facilitate more efficient reactivation of residual language networks. Furthermore, RSF is compatible with basic smartphone functionality (e.g., audio/video recording), making it highly accessible and cost-effective, even in under-resourced or remote contexts. These attributes make RSF an ideal procedure to bridge gaps in aphasia care for minority-language speakers or those without access to language-matched clinicians, as it allows them to rehearse speech using materials in their preferred language and phonetic system.

### 5.2 Personalized aphasia therapy using artificial intelligence

Artificial Intelligence (AI) is revolutionizing aphasia assessment and therapy by enabling more language-specific, culturally adaptive, and personalized interventions (Azevedo et al., 2024). Machine learning models, natural language processing, and automated speech analysis enhance diagnostic accuracy, predict recovery trajectories, and customize treatment strategies. Studies have shown that AI-powered applications can make aphasia treatment more precise, data-driven and evidence-based (Azevedo et al., 2024; Medenica et al., 2024).

Computerized interventions, such as virtual reality (VR), create immersive, gamified environments that engage patients and simulate real-world communication challenges, enhancing motivation and language practice (Cao et al., 2021). Beyond language outcomes, these non-linguistic approaches help address cultural, logistical, and linguistic barriers, as well as the shortage of speech-language therapists, making aphasia rehabilitation more accessible (Jacobs et al., 2021). An example is EVA Park, a multi-user virtual environment designed to enable individuals with aphasia to engage with their speech pathologists and connect with other PWA (Galliers et al., 2017). AI-driven speech technologies are increasingly being integrated into aphasia rehabilitation, enhancing accessibility and allowing patients to receive therapy remotely through tablet and computer-based software (Jiang et al., 2024). Innovative technology-based interventions, such as those examined by Repetto et al. (2021) provide opportunities for more intensive practice, increasing treatment intensity and leading to improved language recovery outcomes. Additionally, these interventions offer greater flexibility, enabling patients to select tasks and practice at their own pace, thereby fostering motivation and engagement in therapy (Repetto et al., 2021). In addition to enhancing accessibility and treatment intensity, speech recognition and synthesis technologies such as text-to-speech converters and voice banking systems help PWA improve their expressive language skills (Latif et al., 2021).

While promising, AI is not without limitations. AI systems are prone to generating plausible yet inaccurate outputs, often described as “hallucinations” or, more appropriately, “AI misinformation, which may mislead patients or clinicians if used without oversight (Hatem et al., 2023; Ray and Majumder, 2023). Ensuring clinical accuracy, safeguarding patient data, and incorporating human review mechanisms will be essential to responsibly integrating AI into aphasia care. To realize the full potential of AI and digital tools in aphasia care, it is essential to expand linguistic coverage and incorporate cross-linguistic adaptability into design, including the ability to accommodate code-switching, mixed-language use, and regionally distinct language norms. Addressing these gaps is essential for AI-based interventions to deliver equitable, scalable aphasia therapy for linguistically diverse populations (Privitera et al., 2024).

### 5.3 Multilingual artificial intelligence for universal aphasia therapy

The integration of large language models (LLMs) into aphasia therapy presents promising opportunities for the development of multilingual AI agents capable of delivering individualized interventions across diverse linguistic contexts. LLMs are advanced artificial intelligence systems that leverage deep learning—particularly transformer-based architectures—to model, predict, and generate human language in both monolingual and multilingual formats (Annepaka and Pakray, 2025; Vaswani et al., 2023). Contemporary LLMs are not only multilingual but also multimodal, allowing them to process and generate language across various sensory modalities (e.g., text, speech, image, video). These capabilities make LLMs well-suited and highly promising for supporting aphasia therapy in diverse populations by enabling tailored interventions that span languages and communication modalities.

LLMs have been integrated into the management of neurological conditions such as dementia and psychosis, particularly in diagnosing and predicting language disorders (de Arriba-Pérez and García-Méndez, 2024). Studies have indicated that it could also be integrated into aphasia care by improving language assessment, enabling personalized therapy, and facilitating real-time feedback, ultimately improving communication outcomes for PWA (Themistocleous, 2024; Zhong, 2024). For instance, LLMs have shown potential to detect paraphasic errors, identify agrammatic structures, and refine automatic speech recognition (Cong et al., 2024). Specifically, Cong et al. (2024) conducted a large-scale NLP study examining pre-trained LLMs across English and Mandarin speech samples and found that while English models achieved moderate accuracy in aphasia detection, Chinese models performed poorly, highlighting significant discrepancies in performance across languages. Similarly, a study by van V et al. (2024) reported that applying LLM-based procedures to patients with Broca’s aphasia improved speech corrections and syntactic organization, addressing their characteristic halted and fragmented speech patterns. A recent pilot study described the development and initial testing of Aphasia-GPT, a mobile web app designed to support people with aphasia by providing suggested utterances based on users’ spoken input. Testing with three individuals with moderate aphasia indicated the system currently functions best as a sentence production aid for non-fluent aphasia with relatively preserved articulation, though further improvements are needed to support a wider range of speech profiles and communicative needs (Bailey et al., 2024). These findings highlight the potential of LLMs and AI, particularly when paired with expert-in-the-loop, in revolutionizing aphasia therapy by integrating AI-driven language processing and human expert guidance into clinical practice.

While these studies are encouraging, they also reinforce concerns about equity and accuracy in underrepresented languages. Without sufficient, ethnolinguistically representative training data, even state-of-the-art models may struggle to reliably detect or support therapy in heritage, minority, or low-resource languages. LLMs can misinterpret culturally specific syntax, discourse patterns, or error types—potentially undermining assessment and intervention outcomes (Yari and Koto, 2025; Zhou et al., 2025). Moreover, models may produce plausible but incorrect or misleading outputs if used without clinician validation.

Multilingual conversational AI agents, built using multilingual LLMs, have the potential to adapt to more diverse aphasic errors than previously studied which make them potentially useful for clinical applications. For instance, our research employed a novel approach—*Agent-Based Conversational Dialogue* (ABCD)—to simulate context embedded therapeutic speech interactions between an AI-based therapist and an AI-modeled aphasic patient. The patient agent was designed to vocally imitate characteristic word-and discourse-level errors observed in English-speaking individuals with aphasia, enabling ecologically valid modeling of therapeutic dialogue. The study used GPT4o as the core LLM for both AI-agents and their behaviors were modulated using well-crafted prompts rather than traditional finetuning of pretrained models using large dataset of aphasic speech data. Our findings showed that the AI-therapist’s conversational performance, in terms of context and logically-relevant utterances, were highly accurate despite aphasic speech inputs such as language mixing errors in English and Spanish, tangential utterances, and semantic, phonemic, perseveration and syntactic word errors (Imaezue and Marampelly, 2025). We further replicated the performance of the AI-therapist in an ABCD simulated setting across other languages such as Spanish and Hindi (Imaezue et al., 2024a).

While these preclinical findings are highly promising, further research is needed to test the responsiveness and accuracy of AI-driven therapy and AI-driven therapy plus expert-in-the-loop with cross-linguistic patients with aphasia across diverse quantitative and qualitative metrics. Furthermore, more studies are required to demonstrate the clinical efficacy of these systems, especially administered via smartphones, on aphasia recovery in humans. Notwithstanding, multilingual LLMs and related technologies are still limited to only a few languages. This suggests that its potential clinical applicability is still constrained by linguistic factors.

### 5.4 Ethical and practical considerations

As AI-driven tools and telehealth platforms are increasingly integrated into aphasia therapy, safeguarding patient data has become an essential ethical concern (ElHennawy, 2024). Abujaber and Nashwan (2024) argued that ethical frameworks for AI in healthcare must account for cultural and linguistic diversity at all stages, from data acquisition to algorithmic deployment. By foregrounding these issues, we propose that data privacy and security are not peripheral concerns but essential components of linguistically responsive digital therapy. The storage and transmission of sensitive health information must adhere to stringent data protection regulations to prevent breaches and unauthorized access (Abujaber and Nashwan, 2024). Clinicians and developers must implement encryption, secure authentication protocols, and consent-based data-sharing policies to maintain patient trust and confidentiality. Moreover, the use of AI models trained on patient speech data necessitates strict measures to ensure de-identification and protect individuals’ privacy while enhancing therapy effectiveness (Medenica et al., 2024). Failure to address these privacy concerns may undermine the widespread adoption of these technologies, particularly in culturally sensitive contexts where trust is paramount (Marks and Haupt, 2023).

### 5.5 Training and resource allocation

As multilingual and AI-assisted therapies continue to gain traction, there is a growing need for clinician training in both technological and linguistic competencies. SLPs must be equipped with the skills necessary to utilize AI-driven diagnostic tools, telehealth platforms, and multilingual therapy strategies effectively (Azevedo et al., 2024). Collaboration with interdisciplinary teams, including neurologists, psychologists, computer scientists and software developers, is essential in refining these interventions and ensuring their effective integration into clinical practice (Hinckley and Jayes, 2023). Furthermore, resource allocation is vital to support the development of culturally inclusive therapy programs, which can help bridge existing treatment gaps in multilingual aphasia care (Jacobs and Ellis, 2025). By investing in these areas, the healthcare system can ensure that new technologies for aphasia and related disorders are not only innovative but also accessible and relevant to diverse cross-linguistic patient populations.

## 6 Conclusion

In conclusion, this critical review underscores the evolving landscape of aphasia research in culturally and linguistically diverse populations, highlighting both traditional and emerging restorative treatment approaches. Although progress has been made in addressing multilingual contexts, further research is essential to advance neurobiologically grounded restorative therapies as primary treatment modalities for diverse language speakers. There is also a growing need to explore mobile health solutions and develop multilingual, multimodal AI-based therapies tailored to multicultural communities. Expanding these areas will support the creation of more inclusive, effective, and culturally responsive aphasia interventions—ultimately paving the way towards universal aphasia care.
